# In-lab synthesized turn-off fluorescence sensor for estimation of Gemigliptin and Rosuvastatin polypill appraised by Spider diagram, AGREE and whiteness metrics

**DOI:** 10.1038/s41598-024-53203-z

**Published:** 2024-02-05

**Authors:** Sara M. Mohyeldin, Wael Talaat, Miranda F. Kamal, Hoda G. Daabees, Mohsen M. T. El-Tahawy, Reda M. Keshk

**Affiliations:** 1 Department of Pharmaceutical Analytical Chemistry, Faculty of Pharmacy, Damanhour University, Damanhour, Egypt; 2 Department of Pharmaceutical Chemistry, Faculty of Pharmacy, Damanhour University, Damanhour, Egypt; 3 Department of Chemistry, Faculty of Science, Damanhour University, Damanhour, Egypt

**Keywords:** Green chemistry, Analytical chemistry, Fluorescent probes, Sensors

## Abstract

Gemigliptin-Rosuvastatin single-pill combination is a promising therapeutic tool in the effective control of hyperglycemia and hypercholesterolemia. Organic sensors with high quantum yields have profoundly significant applications in the pharmaceutical industry, such as routine quality control of marketed formulations. Herein, the fluorescence sensor, 2-Morpholino-4,6-dimethyl nicotinonitrile **3**, (λex; 226 nm, λem; 406 nm), was synthesized with a fluorescence quantum yield of 56.86% and fully characterized in our laboratory. This sensor showed high efficiency for the determination of Gemigliptin (GEM) and Rosuvastatin (RSV) traces through their stoichiometric interactions and simultaneously fractionated by selective solvation. The interaction between the stated analytes and sensor **3** was a quenching effect. Various experimental parameters and the turn-off mechanism were addressed. The adopted approach fulfilled the ICH validation criteria and showed linear satisfactory ranges, 0.2–2 and 0.1–1 μg/mL for GEM and RSV, respectively with nano-limits of detection less than 30 ng/mL for both analytes. The synthesized sensor has been successfully applied for GEM and RSV co-assessment in their synthetic polypill with excellent % recoveries of 98.83 ± 0.86 and 100.19 ± 0.64, respectively. No statistically significant difference between the results of the proposed and reported spectrophotometric methods in terms of the *F*- and *t*-tests. Ecological and whiteness appraisals of the proposed study were conducted via three novel approaches: the Greenness Index via Spider Diagram, the Analytical Greenness Metric, and the Red–Green–Blue 12 model. The aforementioned metrics proved the superiority of the adopted approach over the previously published one regarding eco-friendliness and sustainability. Our devised fluorimetric turn-off sensing method showed high sensitivity, selectivity, feasibility, and rapidity with minimal cost and environmental burden over other sophisticated techniques, making it reliable in quality control labs.

## Introduction

Dyslipidemia is a highly prevalent risk factor with alarming morbidity and mortality effects in those suffering from diabetes mellitus (DM). The presence of insulin resistance or deficiency offsets the homeostatic balance of the plasma lipoproteins. This is characterized by elevated levels of triglycerides (TG), and low-density lipoprotein cholesterol (LDL-C), along with reduced levels of high-density lipoprotein cholesterol (HDL-C)^1^. These metabolic changes, in turn, constitute central risk elements for the process of atherogenesis and cardiovascular disease (CVD). Consequently, hyperinsulinemia and elevated cholesterol levels serve as key treatment targets for diabetic dyslipidemia, achieved via fixed combination therapy involving oral antidiabetic and statin^2,3^.

The single polypill merging Gemigliptin and Rosuvastatin, Zemiro (50/20mg), is a promising therapy for the management of uncontrolled hyperglycemia and hypercholesterolemia. Despite both drugs being effective as monotherapy, the fixed-dose combination has the added value of reduced pill burden, enhanced patient compliance, and long-term benefits^4,5^.

**Figure 1:**
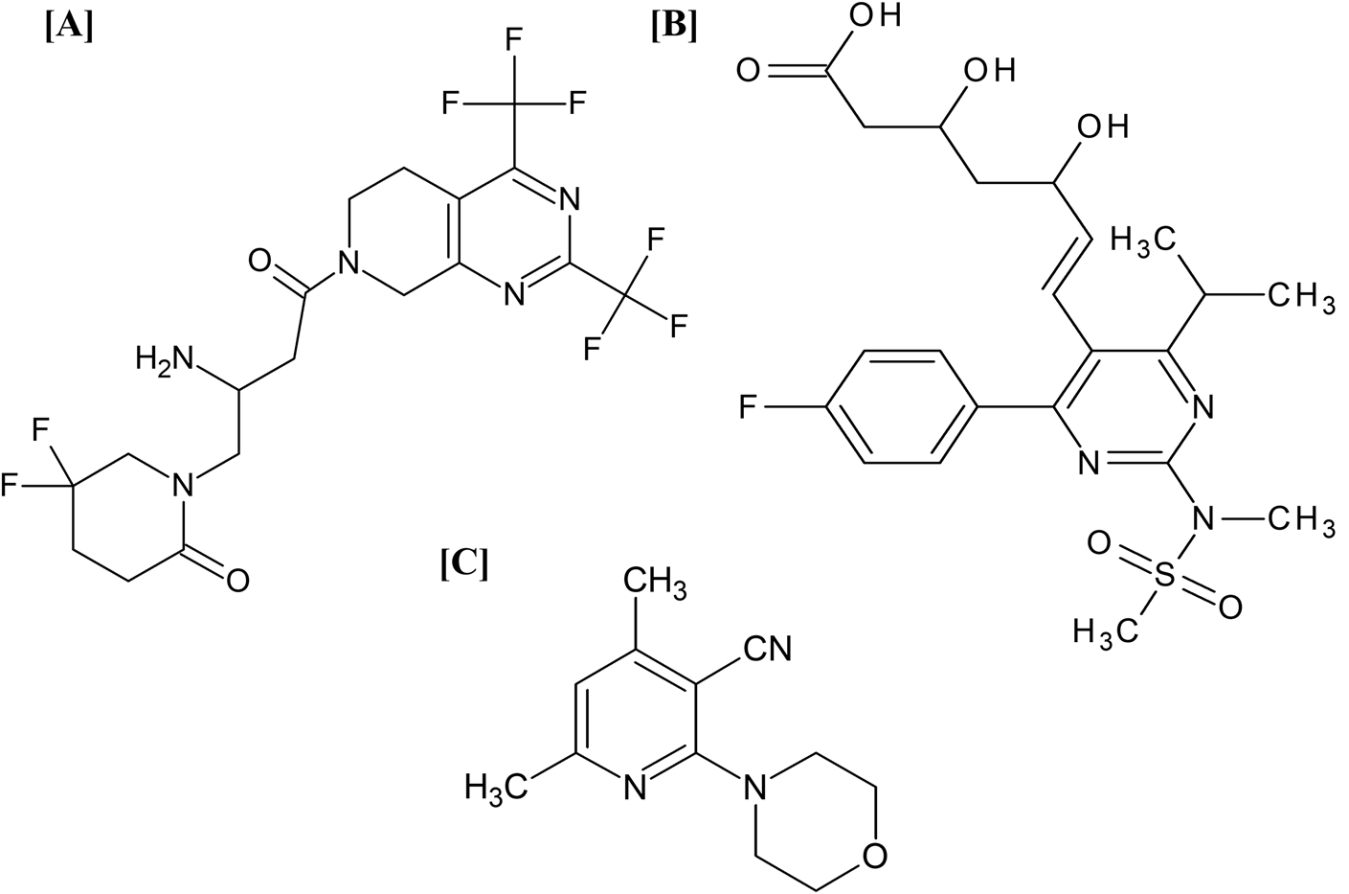
Chemical structures of (**A**) Gemigliptin, (**B**) Rosuvastatin, and (**C**) Fluorescence sensor.

Gemigliptin (GEM) is one of the latest developed long-acting competitive dipeptidyl peptidase-4 (DPP-4) inhibitors with selective action and high potency^6^. It is chemically known as (3S)-3-amino-4-(5,5-difluoro-2-oxopiperidino)-1-[2,4-di(trifluoromethyl)-5,6,7,8-tetrahydropyrido[3,4-d] pyrimidin-7-yl]butan-1-one (Fig. 1A). It exerts its action through inhibition of the DPP-4 enzyme, with a consequent increase in endogenous incretin hormones. This improves glucose-dependent insulin secretion and postprandial hyperglycemia while diminishing pancreatic glucagon secretion. Additionally, GEM has shown some favorable effects on triglyceride and total cholesterol levels in diabetic patients^7,8^.

Rosuvastatin (RSV) is the most potent fully synthetic member of statins. It is chemically defined as (E,3R,5S)-7-[4-(4-fluorophenyl)-2-[methyl(methylsulfonyl)amino]-6-propan-2-ylpyrimidin-5-yl]-3,5-dihydroxyhept-6-enoic acid (Fig. 1B). It is a competitive inhibitor of 3-hydroxy-3-methyl-glutaryl coenzyme-A (HMG-CoA) reductase with characteristically limited extrahepatic activity. This allows an effective decline in LDL-C and overall cholesterol levels, as well as an elevation in HDL-C levels, with a significant CVD risk reduction. RSV’s efficacy is largely attributed to its sulfonyl group, which renders it more hydrophilic and hence a unique member of statins^9,10^.

A thorough literature review revealed only one reported approach for concurrently assessing GEM and RSV in their binary mixture using spectrophotometry^11^ which, despite its availability and cost-effectiveness, lacks sufficient sensitivity. Conversely, chromatographic methods, including HPLC and GC, offer superior sensitivity; however, their limited time and cost efficiency, as well as the demand for considerable amounts of harmful organic solvents, sophisticated equipment, and procedures, impede their widespread application^12^. Spectrofluorimetric analysis has the combined advantages of being highly sensitive, easily operable, and less expensive, with the ability to analyze multiple samples in a shorter time duration with significantly less solvent use^13^. These merits promote its use in quality control labs instead of other laborious analytical techniques. To the extent of our knowledge, no fluorometric assay has been reported for the co-evaluation of both stated drugs in their fixed-dose pill. This prompted us to adopt the fluorimetric turn-off sensing-based analysis.

Over the past decades, fluorescence sensors have undergone significant development, proving to be a versatile option in the field of analytical chemistry^14,15^. The current interest in these sensors is backed by a multitude of merits, including their sensitivity, selectivity, minimal molecular impact, cost and time effectiveness without being unduly complicated^16^. Its positive potential sets fluorescence sensors as a promising tool with significant applications in pharmaceutical and biological studies^17,18^. This has led many researchers worldwide to embrace these sensors for analyzing a variety of drugs in their single^19–21^ or multi-component forms, either by solubility-^22^ or pH-dependent selective extraction^23^.

In light of the above-mentioned information, the current study seeks to design, for the first time, a selective, effortless, sustainable, as well as ecologically benign fluorimetric quenching-dependent approach for the precise quantification of GEM and RSV in their pure and fixed-dose forms. To achieve our goal, a new fluorescence sensor was synthesized in our laboratory via a simple preparative method. Physicochemical characterization and stability-assuring forms of the synthesized fluorophore were thoroughly performed prior to the drugs’ analyses. Moreover, smart solvent choice aids the selective discrimination and simultaneous appraisal of the stoichiometric quenching reactions of both GEM and RSV with the synthesized sensor. Lastly, our adopted method was meticulously optimized, validated, and applied to raw materials as well as pharmaceutical formulation.

Ecological approval remains a routine challenge for novel analytical procedures. Accordingly, the suggested method is compared with the reported spectrophotometric one for its ecological toll and whiteness via the recently released appraisal metrics: Greenness Index via Spider charts, Analytical Greenness (AGREE), and Red–Green–Blue (RGB) 12.

## Experimental

### Instrumentation

Regarding the synthesized fluorophore’s physicochemical properties, the melting point was determined using the Mel-Temp II equipment. IR spectra (KBr) were measured using a Perkin Elmer FT/IR spectrophotometer (Alexandria University). ^1^H NMR and ^13^C NMR spectra were captured on a JEOL (500 MHz) utilizing tetramethyl-silane (TMS) as an internal reference standard and DMSO d_6_ as a solvent (El-Mansoura University). In the Microanalytical Unit of Cairo University, the elemental analysis (C, H, and N) was measured. For spectrofluorimetric analysis, an Agilent Cary Eclipse fluorescence spectrophotometer (USA), outfitted with a 150-W xenon lamp and a 1-cm quartz cell, was utilized. Both the excitation and emission monochromators' slit widths were tuned to 1.5 nm. The apparatus calibration was routinely examined with reference quinine sulphate (0.01 μg/mL) at excitation and emission wavelengths of 250 and 455 nm, respectively. pH measurements were conducted using a 3310 Jenway digital pH meter.

### Materials and reagents

Gemigliptin (GEM) (CAS Number: 911637-19-9) was purchased from Shanghai Jinhe Biotechnology Co. Ltd, China. Rosuvastatin (RSV) was generously supplied by Borg Pharmaceutical Industries, Alexandria, Egypt. Analytical-grade chloroform, isopropanol, sodium dihydrogen phosphate, disodium hydrogen phosphate, orthophosphoric acid, and sodium hydroxide were procured from El-Nasr Chemical Co., Egypt. Tween 80 (T80), cetrimide (CTAB), as well as sodium dodecyl sulphate (SDS) were sourced from Sigma Aldrich (St.Louis, MO, USA). Acetonitrile and methanol were both of HPLC grade (Fisher, UK). Ultra-purified water was used throughout the entire study. For GEM/RSV fixed-dose pills, laboratory-formulated tablets encompassing 50 mg GEM and 20 mg RSV as in Zemiro, along with magnesium stearate, starch, lactose monohydrate, and aerosol (El-Nasr Pharmaceutical Chemicals Co, Egypt), were analyzed.

### Synthesis of 2-Morpholino-4,6-dimethyl nicotinonitrile 3

A mixture of 2-chloro-4,6-dimethyl nicotinonitrile (1.66 g, 0.01 mol) and morpholine (1.7 g, 0.02 mol) was refluxed for 2h in absolute ethanol (30 mL), then left to cool and the formed precipitate was filtered off, dried, and recrystallized from ethanol as white crystals; Yield: 93%; m.p.: 100–102 °C (lit. m.p. 101–103 °C^24^); IR (KBr) cm^−1^: 3059 (CH arom.), 2964, 2922, 2880, 2847 (C–H aliphatic), 2210 (C≡N), 1583 (C=C), 1559 (C=N); ^1^H NMR (DMSO-d_6_) δ ppm: 2.33 (*s*, 6H, two CH_3_), 3.46–3.48 (*t*, 4H, -CH_2_-N), 3.67–3.69 (*t*, 4H, CH_2_-O), 6.77 (*s*, 1H, pyridine-H); ^13^C NMR (DMSO-d_6_) ppm: 19.99 (CH_3_), 24.40 (CH_3_), 48.68 (2C attached to N of morpholine), 65.94 (2C attached to O of morpholine), 116.95 (C≡N), 92.99, 116.30, 154.07, 160.44, 161.18 (pyridine Cs); Anal. Calcd for C_12_H_15_N_3_O (217): C, 66.36; H, 6.91; N, 19.35. Found: C, 66.22; H, 7.15; N, 19.30.

### Preparation of stock solutions

Separate standard solutions of the fluorescence sensor 2-Morpholino-4,6-dimethyl nicotinonitrile **3**, GEM, and RSV (100 μg/mL) were obtained via dissolving 10 mg of each in 100 mL methanol, deionized water, and acetonitrile, respectively. All solutions were kept at 4 °C, away from any light exposure.

### Construction of standard curves

Standard curves were put up for GEM and RSV analysis by correlating the differences in fluorophore emission intensity versus the aforementioned quenchers’ concentrations. The intensity was assessed prior to and after the drugs were added to the synthesized sensor. Using a calibrated 10 mL flask, 0.2 mL of the fluorophore initial solution was combined with 0.1 M SDS volume of 1.5 mL, then diluted to the desired level with deionized water. The latter solution intensity was evaluated at 406 nm upon its excitation at 226 nm. Separate amounts of the GEM and RSV standard solutions were placed into two sets of volumetric 10 mL flasks, followed by 0.2 mL of the sensor and 0.1 M SDS volume of 1.5 mL. Finally, the resulting solutions were diluted with deionized water to achieve concentration levels of 0.2–2 as well as 0.1–1 μg/mL for GEM and RSV, respectively. Similarly, after applying excitation at 226 nm, the emission intensities were detected at 406 nm. The regression equations were deduced after plotting the calibration graphs.

### Analysis of synthetic tablets

The Egyptian medicinal market is devoid of any commercial fixed-dose pills for GEM and RSV. The proposed approach was thus applied to laboratory-generated tablets (comprising 50 mg GEM and 20 mg RSV per tablet as in Zemiro**,** as well as magnesium stearate, aerosol, starch, and lactose as additives). Ten synthetic pills were weighed, properly ground, and blended well. An aliquot of 10 mL acetonitrile was added to exactly one pill’s weight of the powder. The solution was filtered into a 25 mL standardized flask after being sonicated for 10 min. Following two acetonitrile washes of the residue, the resultant filtrate was filled up to the desired level with the latter and marked for RSV evaluation. Regarding the GEM assay, the residue was reextracted with 25 mL deionized water, sonicated for 10 min, then filtered into a calibrated 50 mL flask, the residue was washed twice and finally diluted with water to the required level. After the addition of individual aliquots to fluorophore and 0.1 M SDS, the procedure was performed as previously outlined. The nominal concentrations were computed using either calibration curves or related regression equations.

## Results and discussion

### Synthesis of fluorescence sensor 3

**Figure 2:**
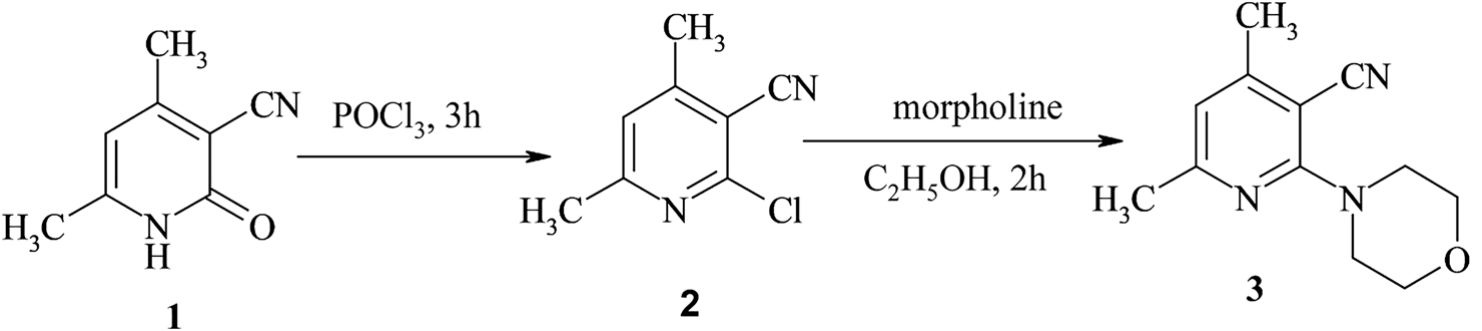
Synthesis schematic diagram of 2-Morpholino-4,6-dimethyl nicotinonitrile sensor **3**.

2-Morpholino-4,6-dimethyl nicotinonitrile **3** (Fig. 1C) was prepared according to the reported procedure^24^. Figure 2 depicts a synthesis schematic diagram of sensor **3**. Its IR spectrum showed the appearance of characteristic absorption bands at 2964, 2922, 2880, and 2847 cm^−1^ for C-H aliphatic, as well as the absorption band at 2210 cm^−1^ for C≡N (Fig. S1). On the other hand, the ^1^H NMR spectra of sensor **3** exhibited two triplet signals at δ 3.46–3.48 and 3.67–3.69 ppm (4H each) attributed to morpholine aliphatic protons, the pyridine proton resonated at 6.77 ppm (Figs. S2, S3). Furthermore, ^13^C NMR of compound **3** showed two signals at δ 19.99, 24.40 ppm for two CH_3_ carbons, and two signals at δ 48.68 and 65.94 ppm (2C each) for morpholine carbons. Cyanocarbon resonated at δ 116.95 ppm, while pyridine carbons resonated at δ 92.99, 116.30, 154.07, 160.44, and 161.18 ppm (Fig. S4).

### Experimental variables influencing the fluorescence

**Figure 3:**
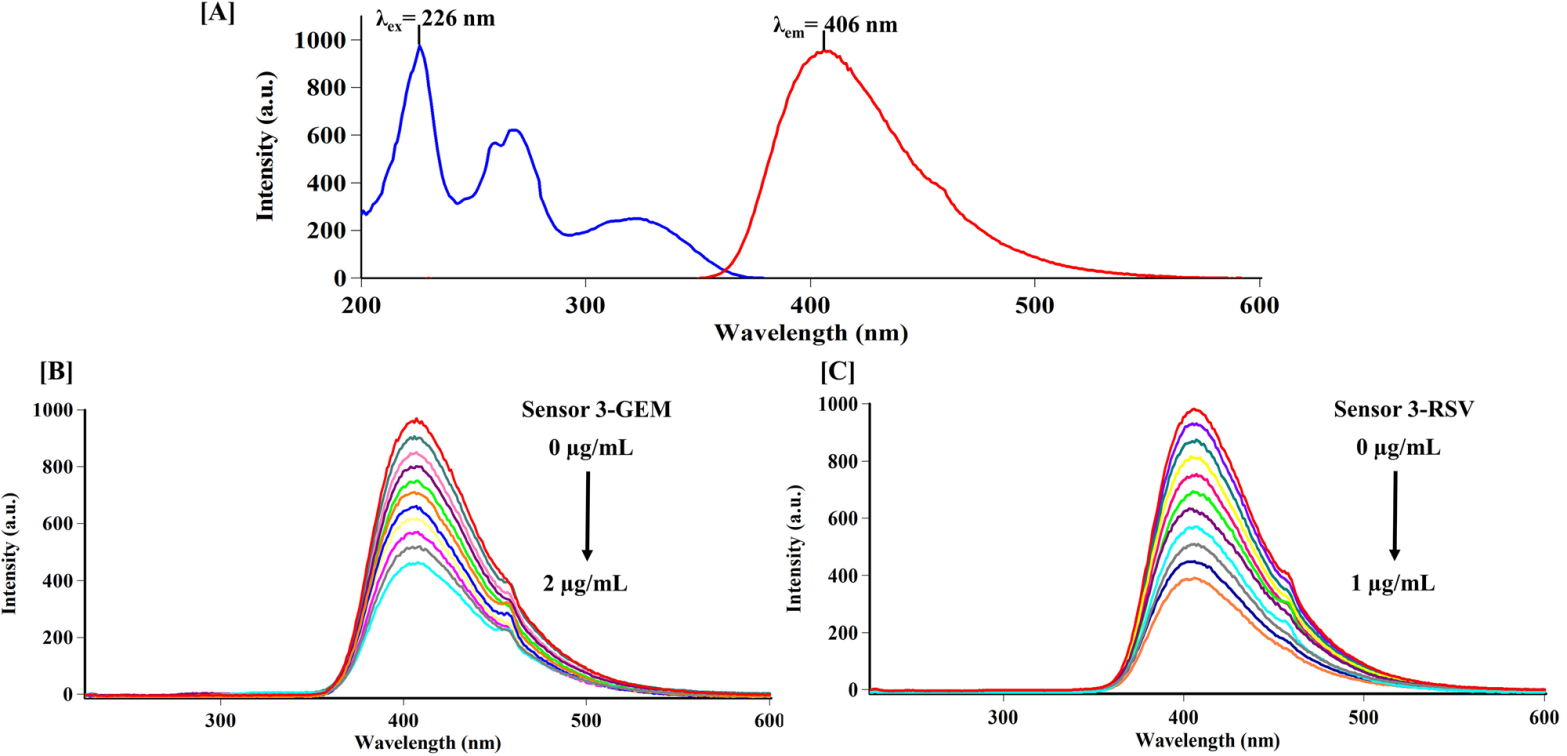
(**A**) Excitation and emission spectra of the fluorescence sensor, and Fluorescence titration spectra of sensor **3** with different concentrations of (**B**) Gemigliptin (0.2–2 μg/mL) and (**C**) Rosuvastatin (0.1–1 μg/mL) in deionized water at 25 °C (λ_ex_ = 226 nm, and λ_em_ = 406 nm).

A new fluorescence sensor was developed in our laboratory, then used in the concurrent appraisal of GEM and RSV in their bulk and co-formulated polypill. As depicted in Fig. 3A, the synthesized fluorophore revealed inherent fluorescence emission at 406 nm upon its excitation at 226 nm. The interaction of the analytes under study with the fluorescence sensor appeared to have quenching effects, as illustrated in Fig. 3B,C.

A variety of experimental parameters that impact both the sensor's fluorescence intensity and the drugs’ quenching effects were investigated and optimized. These parameters include the dilution solvent types, medium’s pHs, types and concentrations of the surfactants, fluorophore concentrations, reaction time, and quantum yield.

#### Impact of diluting solvent

**Figure 4:**
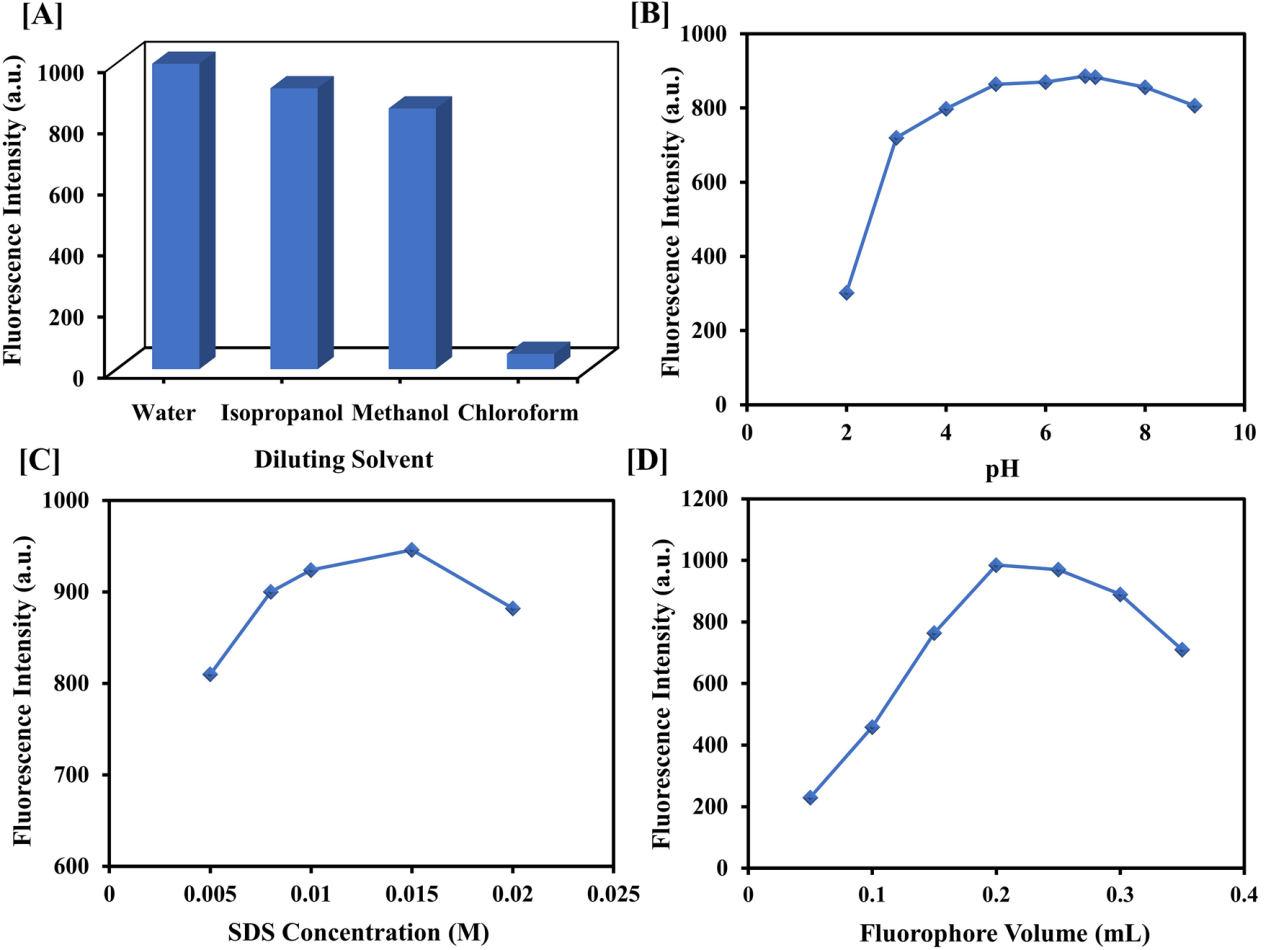
The impact of different (**A**) Diluting solvents, (**B**) pHs, (**C**) SDS concentrations, and (**D**) Fluorophore volumes on the fluorescence intensity of the sensor.

Different polarity solvents, among which deionized water, isopropanol, methanol, as well as chloroform were tried to determine the one that gave the maximum fluorescence intensity (Fig. 4A). Deionized water was observed to be the optimal solvent to achieve maximum sensitivity. This could be ascribed to the fact that higher solvent polarity diminishes the π-π* energy transition while increasing the n-π* energy transition owing to polarity-induced stabilization of the excited state, which has more momentum than the ground state. As a result, the excited-state electron populations increase, ultimately leading to enhanced fluorescence intensity^25^. On the other hand, chloroform’s chloro-substituents are responsible for quenching fluorescence by facilitating intersystem crossing^26^.

#### Impact of pH

The medium’s pH has a significant impact on reaction sensitivity. Using phosphate buffer, the influence of pH on sensor **3** fluorescence was investigated throughout the pH range (2–9) (Fig. 4B). In addition, the pH of the original solution was determined to be 6.8. It was noted that the emission intensity gradually increased with an increase in the pH, reaching a maximum at 6.8, matching the original solution’s pH. Upon further increase in the pH, the fluorescence started to decrease. This pattern could be attributable to the partial fluorophore protonation at a rather neutral pH while being fully protonated at acidic pHs and deprotonated at alkaline pHs^23^. Thus, the need for a buffer solution was deemed unnecessary.

#### Impact of surfactants

Several types of surfactants were tried to determine their influence on both emission intensity and the quenching effect. Sodium dodecyl sulphate (SDS), Cetrimide (CTAB), and Tween 80 were chosen as anionic, cationic, and nonionic surfactants, respectively. Only anionic surfactant (SDS) considerably improved the emission intensity of the fluorophore. The SDS micelles enhance the fluorophore's solubility and guard against oxygen quenching by restricting its entry to sensor **3** molecules^27^. Additionally, a high-viscosity medium improves fluorescence and reduces external conversion. Moreover, the medium’s increased viscosity limits the sensor’s molecular rotation, thus enhancing the likelihood of alignment between the incident light’s electric field and the molecular momentum vector^28^. Lastly, the anionic nature of SDS serves to improve the drugs’ quenching effect as it stabilizes the sensor’s positive charge after electron transfer to the quenchers^27^. On the contrary, cetrimide being positively charged causes instability of the electron-donating sensor, thereby reducing the fluorescence difference^29^. In tween 80, there was no fluorescence due to hydrogen bonds formed with the fluorophore, which promote electron transfer, resulting in quenching^23^. Various concentrations of SDS were tested, ranging from 0.005 to 0.02 M. The maximum emission intensity and drugs’ quenching effect were obtained at a concentration of 0.015 M (Fig. 4C). The utilized concentrations of SDS, CTAB, and Tween 80 were above their critical micelle concentrations of 0.0082, 0.00388, and 0.014, respectively^30–32^.

#### Impact of fluorophore concentration

The fluorophore’s concentration influence was evaluated utilizing various amounts (0.05–0.35 mL) of 100 μg/mL sensor **3** standard solution. A volume of 0.2 mL was considered optimal as it accorded maximum emission intensity (Fig. 4D). Any subsequent increase in fluorophore volume beyond 0.25 mL led to a decrease in response. This could be ascribed to the inner filter effect, in which highly concentrated solutions cause a non-uniform distribution of light intensity to each molecule^26^.

#### Impact of time on reaction

The relation between time and drugs’ quenching effect was assessed by recording the fluorescence intensities for 30 min at 5 min intervals (Fig. S5). Our data revealed that the highest degree of quenching occurred immediately after preparation, following which the detected fluorescence was maintained.

#### Quantum yield

The quantum yield of the prepared fluorophore was evaluated by comparing it to a reference solution of quinine sulphate in 0.1 M sulphuric acid^33^. Various low concentrations of both reference and fluorophore solutions were prepared, and their absorbances were assessed. Moreover, the emission spectra were measured, and the integrated emission intensities were computed. Two plots of the integrated emission intensities against the absorbances were developed (Fig. S6) and their gradients were deduced.

1 $${\text{Y}}_{{\text{a}}} = {772352}.{\text{9 X}}_{{\text{a}}} + {4317}.{61}\quad {\text{r}} = 0.{9992}$$

2 $${\text{Y}}_{{\text{b}}} = { 8133}0{9}.{\text{6 X}}_{{\text{b}}} \, - {16512}.0{4} {\text{r }} = \, 0.{9995}$$

where Y_a_ and Y_b_ are the integrated emission intensities of quinine sulphate and fluorophore, respectively. X_a_ and X_b_ are the absorbances of quinine sulphate and fluorophore, respectively.

The formula below was used to compute the fluorophore`s quantum yield.

3 $${\Phi }_{x}= {\Phi }_{st}(\frac{{G}_{x}}{{G}_{st}})(\frac{{\upeta }_{x}^{2}}{{\upeta }_{st}^{2}})$$

where the subscripts $x$ and $st$ denote the fluorophore and reference quinine sulphate, respectively. $\Phi$, $G,$ and $\upeta$ indicate quantum yield, the gradient, and the solvent’s refractive index, respectively. Given that ${\Phi }_{st}$= 54%^20^.

Sensor **3** quantum yield was computed by substituting gradient values acquired from formulae (1) and (2) in formula (3), which was 56.86%. Both the synthesized fluorophore and quinine sulphate were highly diluted in the exact solvent, deionized water; thus, their refractive indices were expectedly the same.

### Elucidation of the quenching mechanism

**Figure 5:**
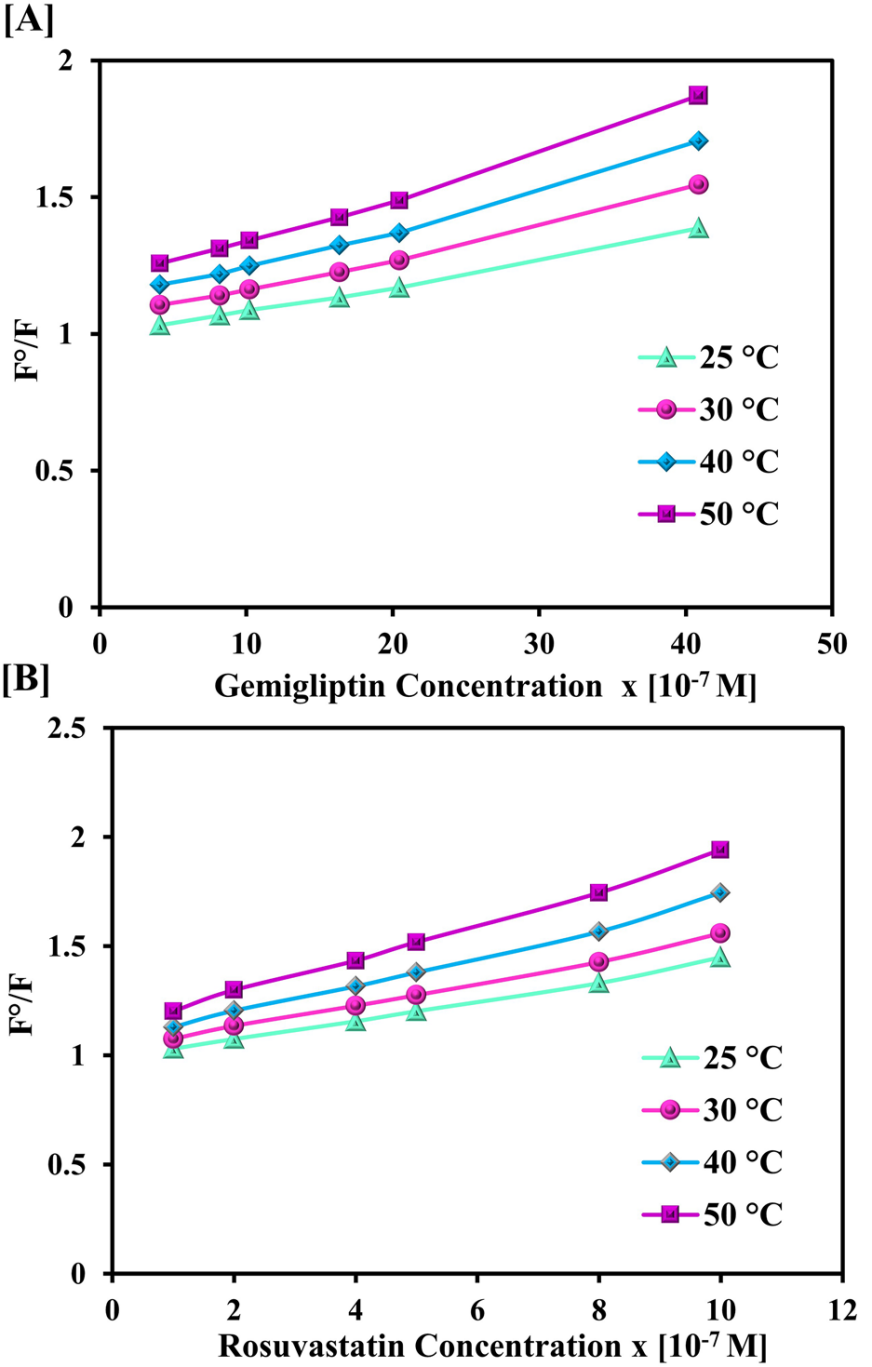
Stern–Volmer plot for the interaction of (**A**) Gemigliptin and (**B**) Rosuvastatin with the fluorescence sensor at different temperatures.

Following the evaluation and refining of the experimental settings, the interactions between the examined drugs, GEM and RSV, as well as sensor **3** were investigated. The apparent quenching impact of both drugs on the fluorophore was detected with a reduction in fluorescence intensity as the GEM and RSV concentrations increased, as shown in Fig. 3B,C, respectively. Additionally, saturation curves for fluorescence titration for the tested drugs were plotted, (Fig. S7). Both analyte interactions were illustrated by Stern–Volmer plots. The ratio between the pre- and post-quenchers fluorophore emission intensities (F^0^/F) was graphed against the quenchers’ molar concentrations [Q] (Fig. 5). The resultant straight lines were suited to the Stern–Volmer equation as follows:

4 $${F}^{0}/F=1+{K}_{sv}\left[Q\right]$$

The relative fluorescence intensity F^0^/F of sensor **3** exhibited an excellent correlation with the content of GEM and RSV (R > 0.996), reflecting the existence of a single type of quenching process, either dynamic or static. Dynamic quenching occurs when the quencher collides with the fluorophore in its excited state. This interaction does not lead to a permanent molecular change. In contrast, a non-fluorescent complex between the fluorophore and the quencher in the ground state is formed during static quenching^29^. To discriminate between both quenching processes, the Stern–Volmer plots were created at different temperatures, 25–50 °C, as depicted in Fig. 5. The slope of the respective plots for both analytes showed a gradual increase with temperature rise, signifying a dynamic quenching effect. This is attributed to the positive relationship between temperature and diffusion rate, with the resultant increase in collisional quenching. While higher temperatures are expected to result in the dissociation of weakly bound complexes and hence reduced static quenching^34^. The Stern–Volmer constants (K_D_) were computed for the dynamic interactions of GEM and RSV; relying on the slopes of Fig. 5. Specifically, K_D_ values for GEM increased from 9.6 × 10^4^ to 16.8 × 10^4^ L mol^−1^ as well as for RSV from 45.6 × 10^4^ to 80.1 × 10^4^ L mol^−1^ as the temperature gradually increased from 25 to 50 °C.

To further verify the type of quenching mechanism induced by GEM and RSV, the UV absorption spectra of the sensor **3,** sensor **3**-GEM, and sensor **3**-RSV systems were scanned (Fig. S8). It was observed that there were no changes in the absorption spectra of sensor **3** before and after the reaction with both analytes. This supports that the synthesized sensor was quenched by GEM and RSV through collisional quenching, which only affects the excited state of the fluorophore^26^.

#### Estimation of the binding constants, binding sites, and Gibb’s free energy

The binding constants (K_b_) and binding site numbers (n) were deduced based on modified Stern–Volmer plots (Fig. S9) according to the double logarithmic formula^35^:

5 $${\text{log}}\frac{{F}^{0}-F}{F} ={\text{log}}{K}_{b}+n{\text{log}}[Q]$$

where F^0^ and F are the emission intensities of the fluorophore in the absence and presence of quenchers, respectively. K_b,_ n, and Q denote the binding constant, the number of binding sites, and the molar concentration of the quenchers, respectively. The K_b_ values were derived from the intercepts (Fig. S9) and found to be 22.01 × 10^4^ for GEM and 29.66 × 10^5^ for RSV. The plotted slopes were 1.07 and 1.14 for GEM and RSV, respectively, indicating a single binding site for the quenching process of both analytes.

Additionally, Gibb’s free energy (ΔG°) was calculated for GEM and RSV using K_b_ values based on the following formula:

6 $$\Delta {G}^{^\circ }=-RT\mathit{ln}{K}_{b}$$

where R is the universal gas constant (8.314 J K^−1^ mol^−1^) and T is the absolute temperature in Kelvin. The calculated ΔG° were − 30.48 and − 36.92 kJ mol^−1^ for GEM and RSV, respectively. The negative values of ΔG° denote that the process is spontaneous and feasible at ambient temperature.

#### Theoretical interpretation of absorption, emission, and quenching of sensor 3

**Figure 6:**
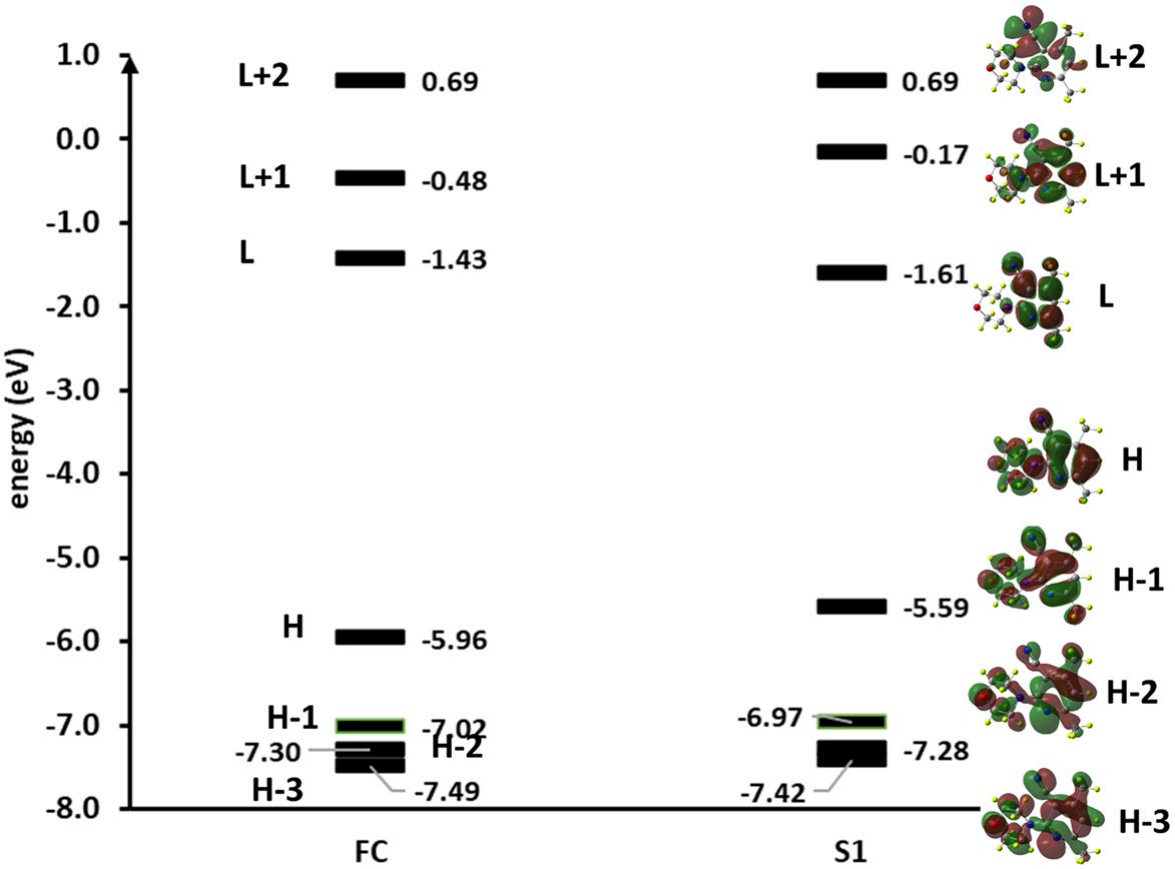
Molecular orbital electron density energies (in eV) were computed for the HOMOs and LUMOs of sensor **3** at the B3LYP/6-61G* level of theory for the ground state (FC) and the lowest excited state (S_1_) geometries. H and L are abbreviated for HOMO and LUMO, respectively.

Geometry optimization in the ground state was carried out using density functional calculations, with the 6-31G* basis sets employing the B3LYP functional. The electronic singlet excited states were calculated using time-dependent density functional theory (TD-DFT) with the same level of theory. The vertical excitation and emission energies and their corresponding oscillator strengths for sensor **3** were computed by applying two different methods. The first method is based on second-order perturbation correction (CASPT2) in the multistate (MS) flavors (MS-CASPT2, hereafter)^36^, thereby using as a reference the complete active space self-consisting field^37,38^ (CASSCF) wave functions averaged over ten roots, while the second method depends on the time-dependent density functional theory (TD-DFT) associated with the B3LYP functional^39^. For both methods, the 6-31G* basis set was used^40^. The water solvent was treated implicitly using the integral equation formalism (IEF) of the polarized continuum model (PCM)^41^ at DFT calculation as implemented in Gaussian 16 package^42^, while MS-CASPT2 was performed in vacuum using OpenMolcas code^43^. The most intensive transitions are reported in Table S1. The molecular orbitals involved in the electronic translations of sensor **3** are displayed in Fig. 6. Generally, both methods of calculation give electronic transitions in good agreement with the experimental values. As shown in Table S1, sensor **3** shows three transitions characterized by increasing values of oscillator strengths and transition energies, with the lowest transition corresponding to the bright first ES (essentially a HOMO (H) → LUMO (L) transition, i.e., π → π*. The H orbital is mainly delocalized on the entire molecule, while the L orbital is localized over the conjugate π-system. Accordingly, this transition involves intramolecular charge transfer to the pyrimidine ring. The experimental peak for this transition is the least intensive one and appears at ca. 320 nm, which is in good matching with the calculated value of 313 nm according to the B3LYP calculation. The medium intense band is shown experimentally at ca. 265 which is red-shifted and blue-shifted with respect to the corresponding calculated values by 13 and 22 nm at B3LYP and MS-CASPT2 levels, respectively. Such a transition is also bright due to the π → π* transition from the H orbital to the L + 1 (Fig. 6). Furthermore, sensor **3** exhibits the strongest intense peak at ca. 226 nm, which is only ± 7 nm off the calculated value. This transition is associated with equal contributions H → L + 2 (41) and H-3 → L (39) corresponding to two different π → π* transitions. According to the oscillator strengths, the most populated state upon excitation is S_6_ (S_5_ according to MS-CAPT2), followed by S_3_ and S_1_, respectively. The higher-populated excited states (S_6_ or S_5_ and S_3_) then relax via the rapid internal conversion to the lowest ES of the fluorophore (S_1_), where they emit to the ground state S_0_. The B3LYP calculation shows that the emission from S_1_ → S_0,_ i.e., L → H, occurs at ca. 365; this value deviates from the observed value by ca. 40 nm; however, employing the more precise MS-CASPT2 method reproduces the experimental value of 406 nm within + 10 red shifting.

Introducing both analytes to the aqueous solution of sensor **3** affects its emission spectra via an energy transfer mechanism. While the higher-energy excited states of the synthesized sensor are populated, energy transfer can occur via the collisional interaction between the excited fluorophore and the quencher molecule before the non-radiative relaxation of the higher excited states through internal conversion to the lowest excited state S_1_ occurs. This is likely to occur due to the large energy separation (ca. 2 eV) between the highly populated excited state (S_5_) and the lowest excited state (S_1_). The overall effect on the emission of sensor **3** is the decrease in fluorescence intensity as the drugs’ concentrations increased.

### Method validation

The validation of the current approach was carried out per ICH recommendations^44^.

#### Linearity and concentration ranges

Under the aforementioned optimal conditions, the adopted approach’s linearity was investigated by analyzing sequential dilutions of each of the tested drugs. Standard curves were created by correlating the differences in sensor **3** emission intensity with the relevant concentrations for each drug. The correlations were linear across the ranges of 0.2–2 as well as 0.1–1 μg/mL for GEM and RSV, respectively. Regression equations were acquired using the least-squares approach to compute correlation coefficients (r), standard deviation of residuals (S_y/x_), intercepts (a), standard deviation of intercepts (S_a_), slopes (b), and standard deviation of slopes (S_b_). Proper linearities were supported by strong correlation coefficients (r ≥ 0.9997), high *F* values, as well as low RSD% of the slope (< 2%). Further statistical parameters, cited in Table 1, were deemed acceptable to satisfy linearity as an essential parameter for validation.

**Table 1:** Analytical parameters of the adopted method.

Parameters	GEM	RSV
Linearity range (μg/mL)	0.2–2	0.1–1
Intercept (a)	22.983	52.167
S.D. of intercept (S_a_)	2.032	3.399
Slope (b)	235.545	510.343
S.D. of slope (S_b_)	1.024	1.936
RSD% of the slope (S_b_%)	0.435	0.379
Correlation coefficient (r)	0.9999	0.9997
R^2^	0.9997	0.9994
S.D. of residuals (S_y/x_)	1.480	1.759
Variance ratio (F)	14,013.2	7872.1
Significance F	3.05 × 10^–8^	9.67 × 10^–8^
LOD (μg/mL)	0.028	0.022
LOQ (μg/mL)	0.086	0.067

#### Limits of detection and quantification

Following the ICH specifications, the limits of detection (LOD) and quantification (LOQ) were computed by applying these formulae: LOD = 3.3 S_a_/b and LOQ = 10 S_a_/b, where S_a_ denotes the standard deviation of the intercepts while b denotes the gradient of the standard plots. As noted in Table 1, the low LOD and LOQ values highlight the sensitivity of the devised approach.

#### Accuracy and precision

In order to ensure the stated method's reliability and consistency, accuracy and precision were examined. For each drug, three separate concentrations were chosen and prepared within the linearity range. Triplicates of samples were evaluated on the same day for accuracy and intra-day precision assessment. This was repeated on three separate days to affirm inter-day (intermediate) precision. For each concentration, recovery%, relative error (Er%), as well as relative standard deviation (RSD%) were computed. Recovery outcomes were satisfactory within the allowable limit of 98–102%. The Er% and RSD% did not surpass 2%, as depicted in Table 2, assuring an outstanding accuracy and precision of the devised approach.

**Table 2:** Accuracy and precision for the determination of the analyzed drugs in bulk form using the presented method.

Parameters	GEM			RSV		
Nominal value (µg/mL)	0.2	1	2	0.1	0.5	1
Intra-day						
Found ± SD	0.199 ± 0.003	0.998 ± 0.012	2.028 ± 0.026	0.1006 ± 0.001	0.506 ± 0.004	0.991 ± 0.008
Recovery%	99.50	99.80	101.40	100.60	101.20	99.10
Er%	− 0.50	− 0.20	1.40	0.60	1.20	− 0.90
RSD%	1.51	1.20	1.28	0.99	0.79	0.81
Inter-day						
Found ± SD	0.203 ± 0.002	1.009 ± 0.015	1.995 ± 0.025	0.1018 ± 0.001	0.504 ± 0.007	1.009 ± 0.009
Recovery%	101.50	100.90	99.75	101.80	100.80	100.90
Er%	1.50	0.90	− 0.25	1.80	0.80	0.90
RSD%	0.99	1.49	1.25	0.98	1.39	0.89

#### Robustness

The current technique's robustness was assessed by applying minor alterations to the chosen variables such as fluorophore volume (0.2 ± 0.02 mL), SDS volume (1.5 ± 0.2 mL), pH (6.8 ± 0.2), λ_ex_ (226 ± 2 nm), and λ_em_ (406 ± 2 nm). The low RSD% (< 2%) revealed that these alterations had no considerable impact on the method’s performance, as stated in Table S2.

#### Selectivity and specificity

**Figure 7:**
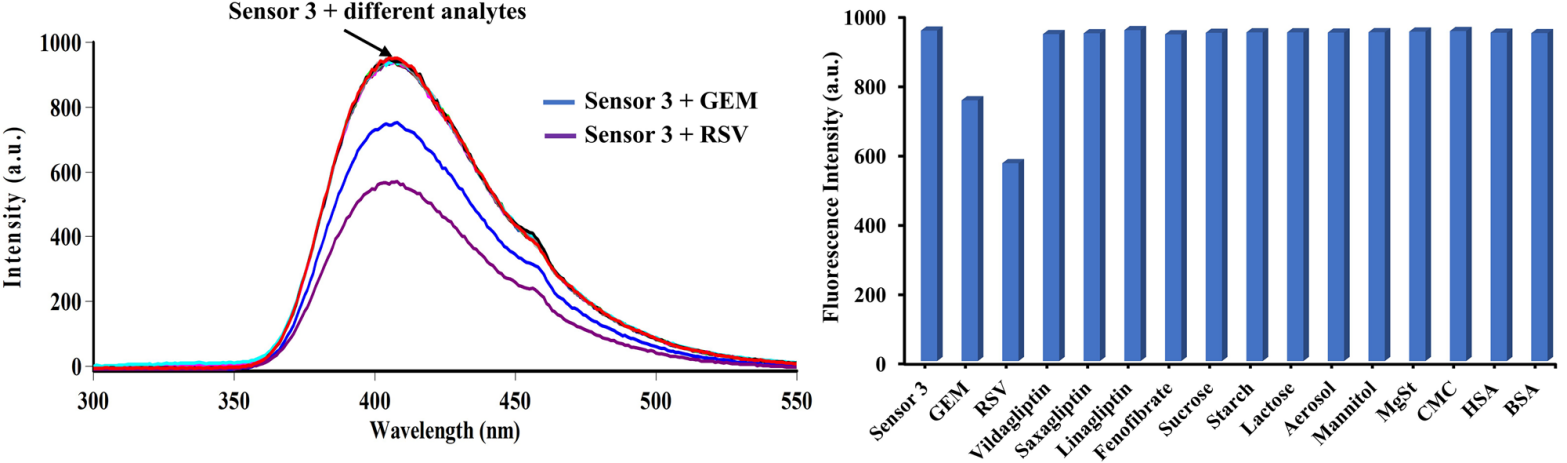
Selectivity and specificity of sensor **3** in the presence of 10 μg/mL of each interference analyte (co-administered drugs, excipients, and serum albumin), Gemigliptin (0.75 μg/mL), and Rosuvastatin (0.65 μg/mL).

The suggested approach’s selectivity was ascertained by assessing the quenching impact of some co-administered drugs, including vildagliptin, saxagliptin, linagliptin, and fenofibrate. These drugs had no quenching effect on sensor **3,** implying no interaction between them and the analyzed ones. Furthermore, the included excipients (sucrose, starch, lactose, aerosol, mannitol, magnesium stearate (MgSt), and carboxymethyl cellulose (CMC)) were tested for their influence on the sensor’s quenching. No impact was observed, confirming the specificity of the adopted method. Human serum albumin (HSA) and bovine serum albumin (BSA) were also investigated in the absence and presence of GEM and RSV, showing no additional inherent quenching impact (Fig. 7).

#### Stability

##### Stability of GEM, RSV, and sensor 3 stock solutions

Two sets of the solutions were prepared: GEM, RSV, and sensor **3** with a concentration of 100 μg/mL for each. The first was stored at ambient temperature, while the second was refrigerated. These solutions were examined hourly for 7 h and subsequently daily for two weeks. The results obtained confirmed the stability of the stock solutions for a week at ambient temperature and 12 days in the refrigerator.

##### Stability of the working solutions

Their stability in deionized water was assessed over two hours at ambient temperature. The emission intensity was found to be maximal at immediate measurement and stayed almost steady over two hours.

### Analysis of raw materials and fixed dosage form

The adopted approach was successfully implemented to analyze GEM and RSV raw materials, as well as laboratory-made polypills. Co-formulated tablets were analyzed by fractional extraction, utilizing a distinct solvent for each drug. Given the different solubilities of both drugs, RSV was selectively extracted by adding acetonitrile, leaving GEM as a residue. Thereafter, deionized water was added to the residue to extract the GEM. The mean % recoveries, standard deviations, as well as % RSD were calculated for both the pure and polypill forms. For the tested drugs, reasonable findings were achieved (Table 3), consistent with the label claim, which demonstrates the absence of influence of the co-existent additives as well as the efficiency of the extraction process. Moreover, the suggested method outcomes were statistically compared with the published spectrophotometric approach^11^ utilizing the *F*- and *t*-tests. The computed values, according to Table 3, didn’t go beyond the tabulated ones, showing that the reported and suggested approaches had no significant differences. Finally, these outcomes reflect that the devised method is applicable for the co-assessment of GEM and RSV in their synthetic fixed-dose pills with reasonable degrees of accuracy, precision, and selectivity.

**Table 3:** Assay of GEM, and RSV raw materials and their combined synthetic tablets using the proposed method and comparison with the reported spectrophotometric method.

	Parameters	Proposed method		Reference method	
		GEM	RSV	GEM	RSV
Raw materials	Mean % recovery ± SD^a^	100.66 ± 0.24	99.51 ± 0.38	100.28 ± 0.53	99.26 ± 0.79
	RSD%	0.24	0.38	0.53	0.80
	*F*-test^b^	0.21	0.23		
	*t*-test^b^	1.47	0.64		
Synthetic Zemiro tablets	Mean % recovery ± SD^a^	98.83 ± 0.86	100.19 ± 0.64	99.77 ± 0.75	99.39 ± 0.94
	RSD%	0.87	0.64	0.75	0.95
	*F*-test^b^	1.25	0.47		
	*t*-test^b^	0.51	1.58		

^a^Mean% recovery ± SD of five determinations.

^b^The tabulated values of *F* and *t*, at p = 0.05, are 6.39 and 1.86, respectively.

### Eco-friendliness and whiteness assessments of the stated method vs. the published one

#### *Greenness index *via* the spider chart metric*

The Greenness index offers a holistic evaluation of the different aspects of the solvents used in our approach and the reported spectrophotometric method^11^. This metric depends on the data extracted from the safety data sheets (SDSs) of the relevant solvents, entailing their different properties and influences on health, safety, and the environment (HSE). As per the GAC postulates, five categories of attributes (Health hazard, General features, Odor, Fire safety, and Stability) are integrated to yield the principal assessment protocols. Each cluster is given a score according to a prespecified algorithm, which ranges from − 5 (least green) to + 5 (most green)^45^. The outcomes are visually displayed as a primary spider chart for the five main criteria, allowing an overview of solvent greenness. Likewise, a more in-depth appraisal of attributes specific to each criterion is presented as secondary spider charts. Several solvents have incomplete SDS sections. For each attribute with missing data, a score of zero is given. All of the criteria are listed in the “Greenness Index Table”, alongside the proportion of accessible data utilized to produce Greenness Index outcomes. This proportion indicates the degree of confidence in the evaluation^46,47^. It’s worth mentioning that this study is among the first to take advantage of the Greenness Index application in the analytical field.

**Figure 8:**
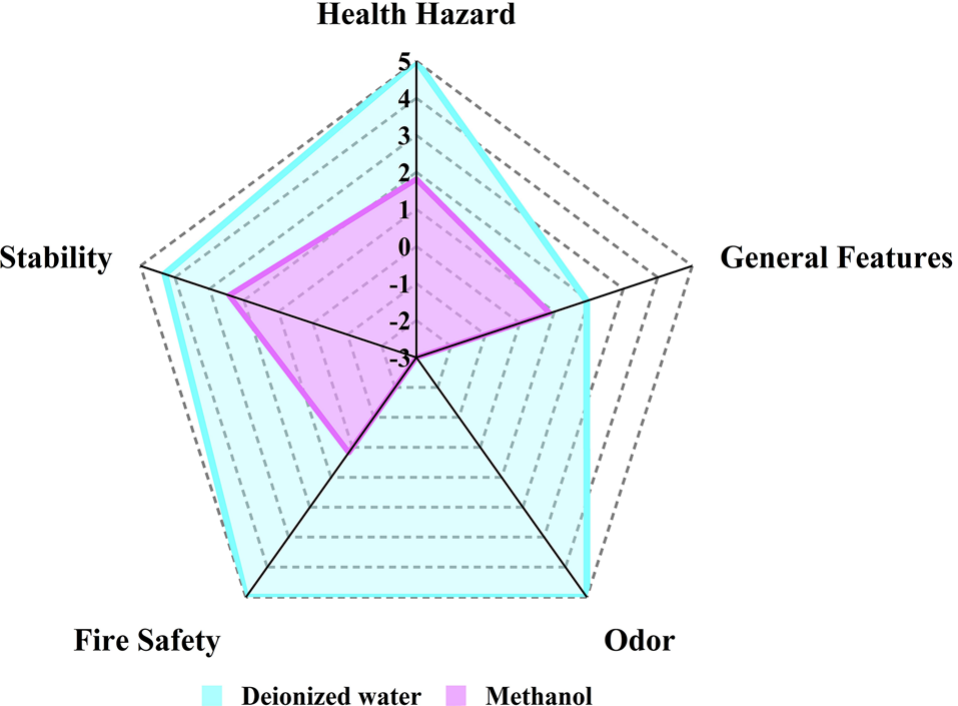
Deionized water and methanol primary spider chart.

**Figure 9:**
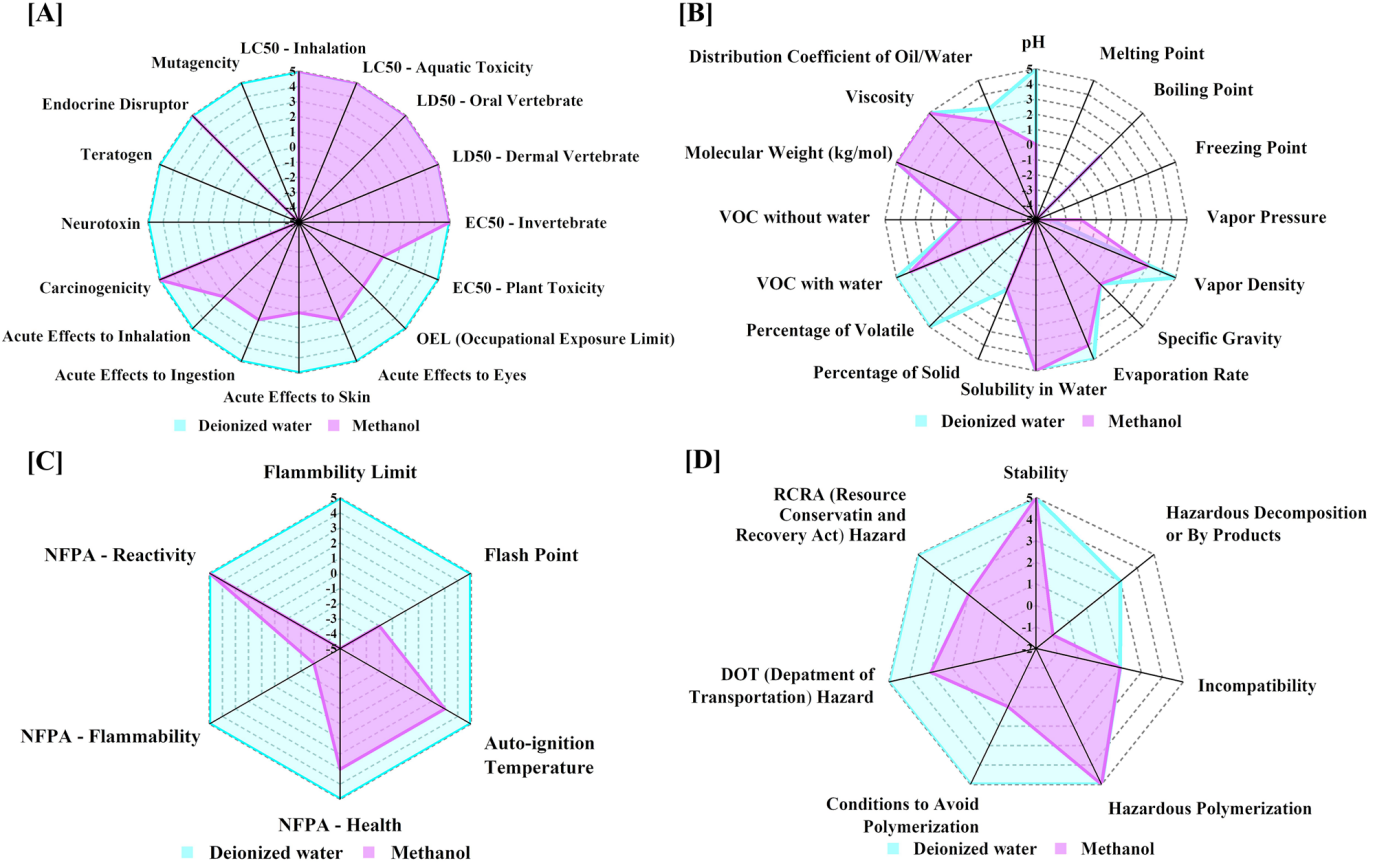
Deionized water and methanol secondary charts for (**A**) health hazard, (**B**) general features, (**C**) fire safety, and (**D**) stability.

This spider approach makes it easy to evaluate a single solvent or compare multiple solvents. In the adopted method, deionized water was used as the solvent, whereas methanol was utilized in the reported spectrophotometric approach^11^. The Greenness Index appraisals for deionized water and methanol are depicted in Figs. 8 and 9, as a primary web chart, and four secondary charts, respectively. The average scores for each criterion, as well as the percentages of data currently available, are exhibited in the Greenness Index table (Table 4).

**Table 4:** Greenness Index table for deionized water and methanol solvents.

Evaluation criteria	Water score	Available information (%)	Methanol score	Available information (%)
Health hazard	5	100	1.81	100
General features	1.94	87.50	0.87	81.25
Odor	5	100	− 3	100
Fire safety	5	100	0.17	100
Stability	4.29	100	2.43	100
Average	4.25	97.50	0.46	96.25

The primary chart, Fig. 8, illustrates that deionized water’s overall greenness index was in the highest safety region, whereas methanol’s lower points placed it in a relatively unsafe zone. The secondary charts, Fig. 9, show a comprehensive evaluation of different aspects of each criterion for the aforementioned solvents. Based on the spider charts and the Greenness Index table data, it can be deduced that the adopted approach is clearly superior to the previously published one as regards human health as well as environmental safety.

#### Analytical greenness metric (AGREE)

This metric provides an extensive and detailed assessment of the approaches’ greenness, along with a ranking of the devised approach and the previously reported one^11^ as per the 12 GAC principles^48,49^. It is an easily downloadable application that generates a simple, colorful pictogram upon inputting twelve variables for each approach. Each pictogram is made up of twelve lateral sectors that range in hue from forest green to red via a central integrated score. The outcome score ranges from zero to one, with values nearer to one, reflecting a greener approach^50,51^. The AGREE model confirmed the superiority of our devised method with a score of 0.86, owing to the use of a naturally abundant, non-toxic, sustainable, and green solvent (water). In contrast, the use of methanol, a toxic inflammable solvent, led to the reported method^11^ being less green, scoring 0.72 (Table 5).

**Table 5:**
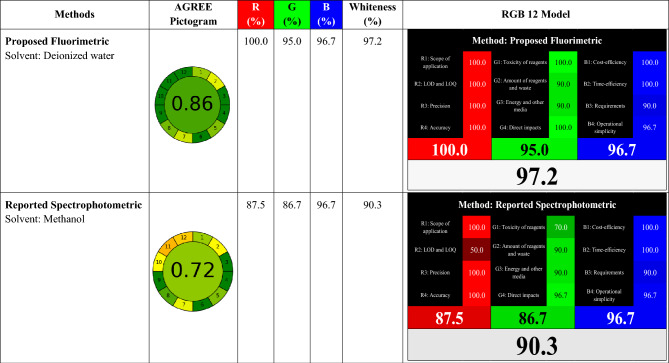
AGREE and RGB12 profiles of the suggested method and the reported one.

#### Whiteness assessment

The Red–Green–Blue (RGB) 12 is a new multifaceted evaluation model that was launched recently. It comprises three complementary aspects, each highlighted with a distinct color: red (analytical validation efficacy), green (eco-friendliness and safety), and blue (economic and practical efficiency). These colors were selected as virtually merging them produces the color white. The RGB 12 design is provided as an Excel template that adheres to WAC standards. It permits up to ten approaches to be concurrently assessed, compared, and evaluated for their sustainability. The results are displayed in the template as a numerical score out of 100, indicating the method's whiteness. Moreover, the obtained chart depicts the percentages of each color specified, with the outcome of their combination, which is whiteness. The most efficient, accurate, and sustainable analytical approach would yield a high overall percentage and be defined as “white”^52,53^.

Both the adopted fluorimetric and the reported spectrophotometric^11^ approaches were objectively and thoroughly compared using the RGB 12 tool. Despite a similar Blue score (96.7%), a discordance was noted in the other colors. As for the Red aspect parameters, the proposed method had a higher score (100 vs. 87.5%), owing to the superior sensitivity as indicated by detection and quantification limits. Furthermore, the use of methanol (3 pictograms) in the published spectrophotometric method^11^ rendered the safety profile toxic, flammable, and health-hazardous. On the contrary, the devised approach used water as a solvent, which reflected in a better Green score (95 vs. 86.7%). As depicted in Table 5 and Fig. S10, the integrated whiteness score for the suggested fluorimetric approach is 97.2%, rendering it a more markedly white method than the reported one of 90.3%.

## Conclusion

Our novel spectrofluorimetric quenching approach presents a facile, economic, and white assessment method for GEM and RSV in their pure form and synthetic polypill. The devised method relied on a new fluorescent sensor synthesized in our lab, the chemical structure of which was verified by spectral data and elemental analysis. The turn-off mechanism was addressed and considered a dynamic quenching for the cited drugs. The presented approach is merited for being more sensitive, selective, cost and time-efficient, thus offering a convenient alternative to other sophisticated techniques. Furthermore, the new method showed an improved level of whiteness and eco-friendliness over the previous one, which was inferred via the Spider charts, AGREE, and RGB 12 assessment tools. It is noteworthy that our study is among the earliest in the analytical field to apply the Greenness Index via spider charts. Considering the above-mentioned benefits, the designed fluorimetric approach is well-suited for routine application in quality control units with a minimal ecological footprint.

### Supplementary Information

Supplementary Information.

## Data Availability

All data will be available upon request. The corresponding author should be contacted for any data required for the conducted study.
