# Supplementary material for: In-lab synthesized turn-off fluorescence sensor for estimation of Gemigliptin and Rosuvastatin polypill appraised by Spider diagram, AGREE and whiteness metrics
Source: Sci Rep. 2024 Feb 5;14:2927. doi: 10.1038/s41598-024-53203-z (PMC10844310; doi:10.1038/s41598-024-53203-z)
Supplement: Supplementary file 1 — Supplementary Information. [file 41598_2024_53203_MOESM1_ESM.docx]

**Supplementary materials**

**In-Lab synthesized turn-off fluorescence sensor for estimation of Gemigliptin and Rosuvastatin polypill appraised by Spider diagram, AGREE and Whiteness metrics**

Sara M. Mohyeldin^1*^, Wael Talaat^1^, Miranda F. Kamal^1^, Hoda G. Daabees^2^, Mohsen M.T. El-Tahawy^3^, Reda M. Keshk^3^

^1^ Department of Pharmaceutical Analytical Chemistry, Faculty of Pharmacy, Damanhour University, Damanhour, Egypt.

^2^ Department of Pharmaceutical Chemistry, Faculty of Pharmacy, Damanhour University, Damanhour, Egypt.

^3^ Department of Chemistry, Faculty of Science, Damanhour University, Damanhour, Egypt.

***** Correspondence: [sara.mohy@pharm.dmu.edu.eg](mailto:sara.mohy@pharm.dmu.edu.eg)

**Figures**

**
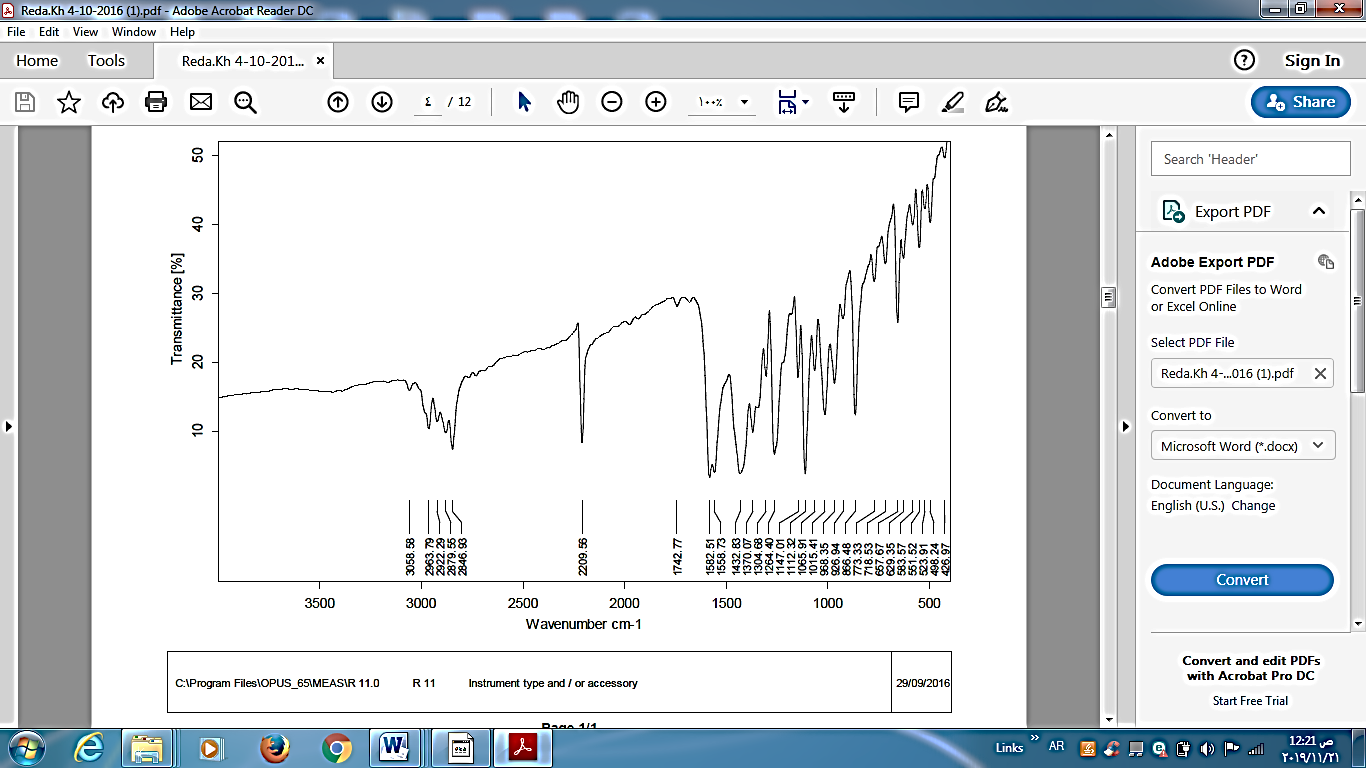
**

**Fig. S1.** IR spectra of sensor **3**

**
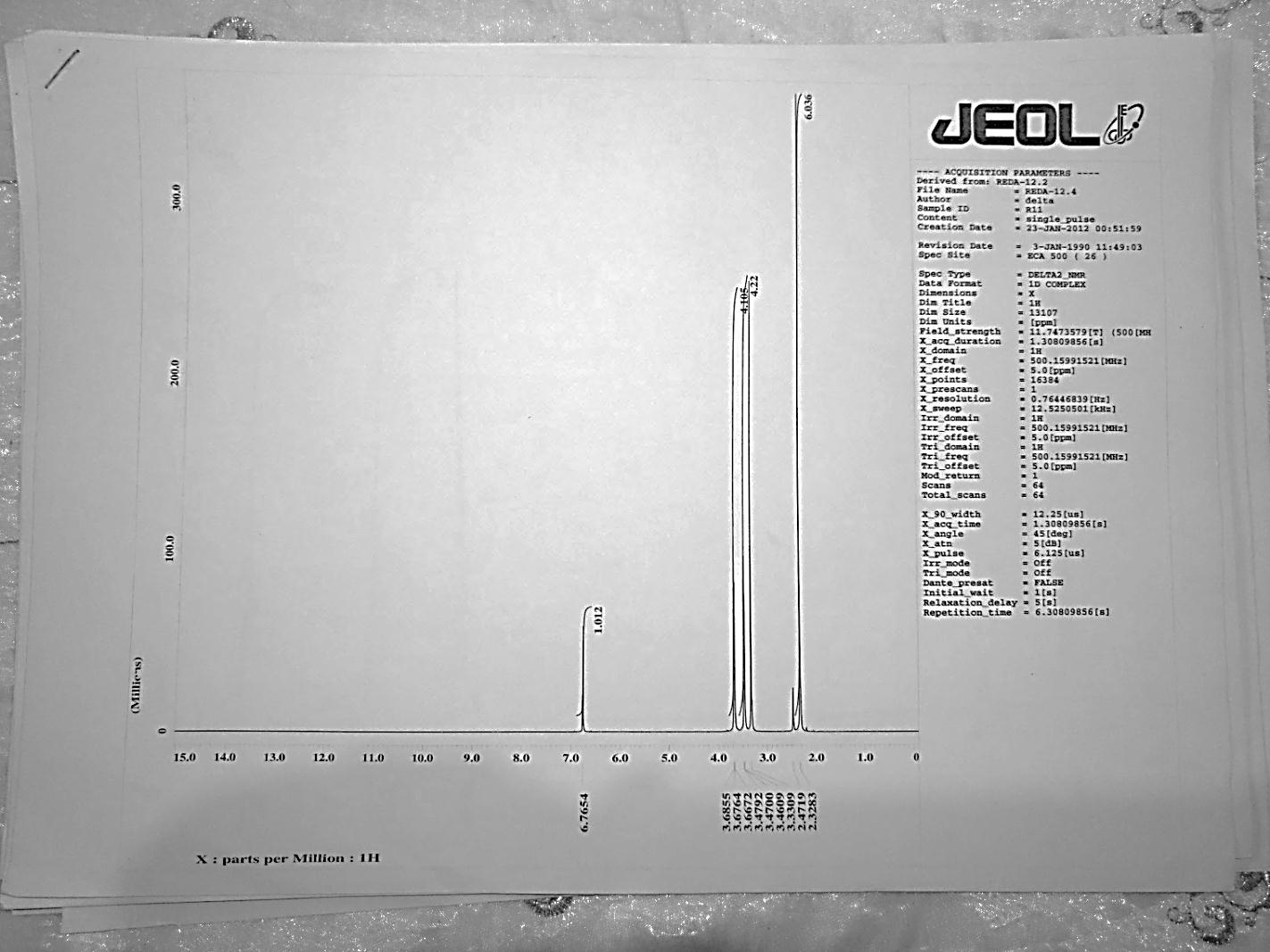
**

**Fig. S2.** ^1^H NMR spectra of sensor **3**


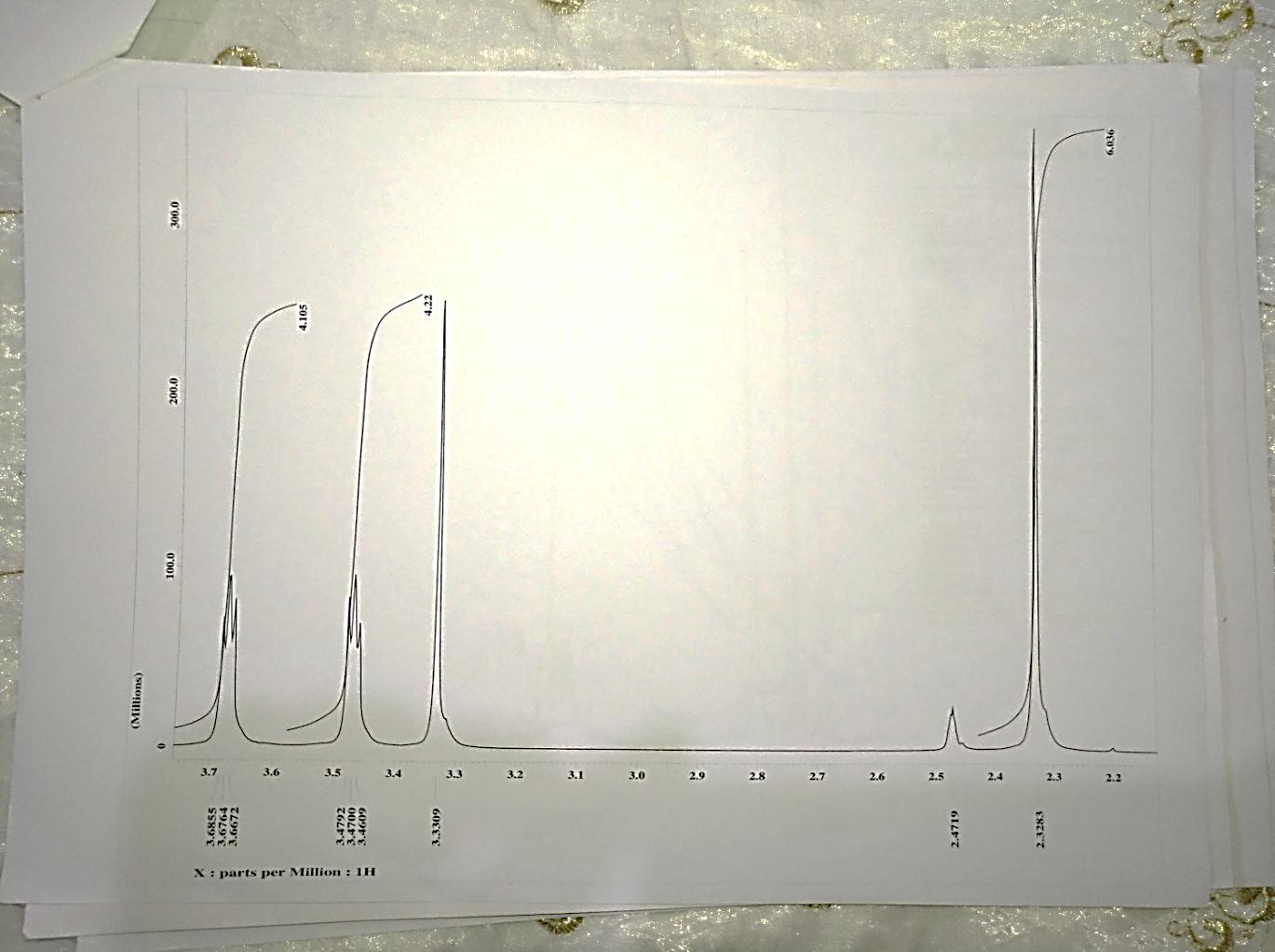


**Fig. S3.** ^1^H NMR spectra of sensor **3**

**
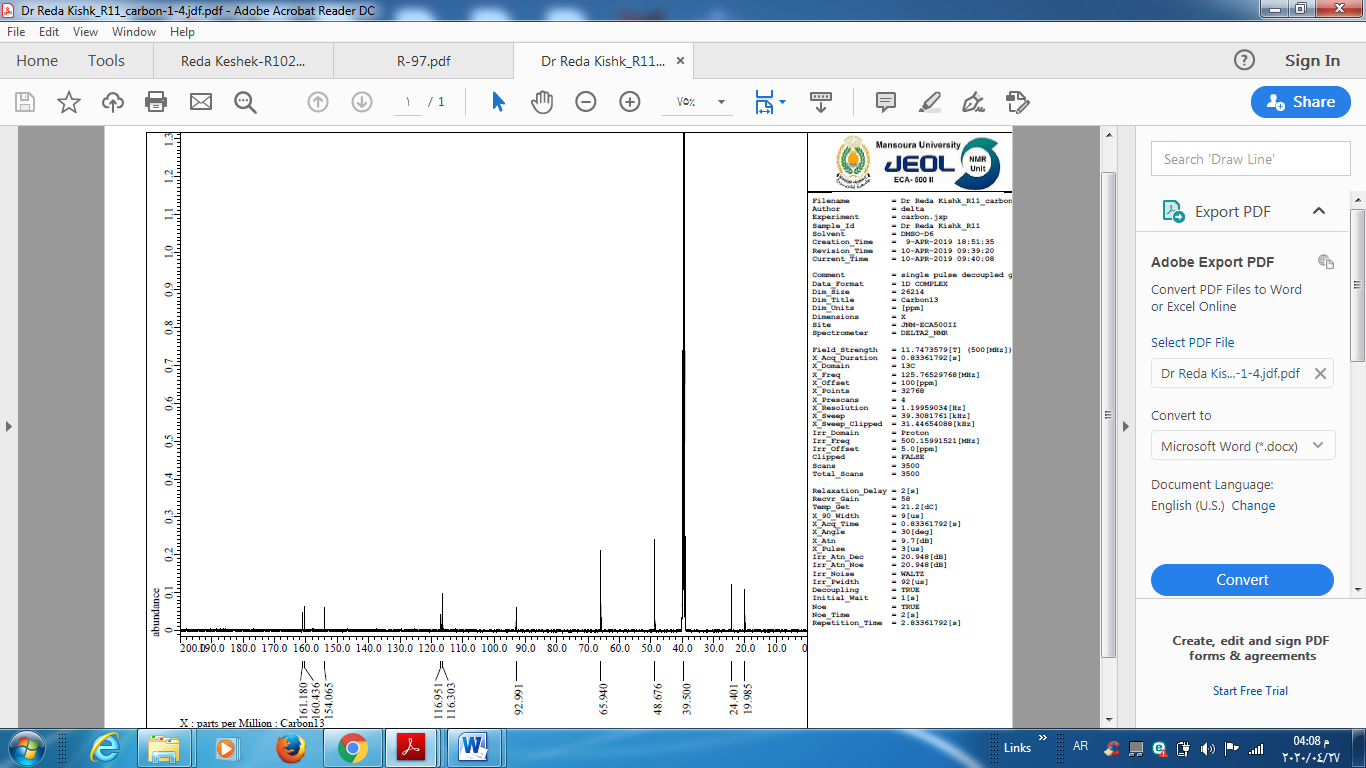
**

**Fig. S4**. ^13^C NMR spectra of sensor **3**

**Fig. S5.** The impact of time on the quenching effect of Gemigliptin and Rosuvastatin over the fluorophore.

**Fig. S6.** A plot of the integrated fluorescence intensity versus the absorbance of standard quinine sulphate, and sensor **3** solutions.


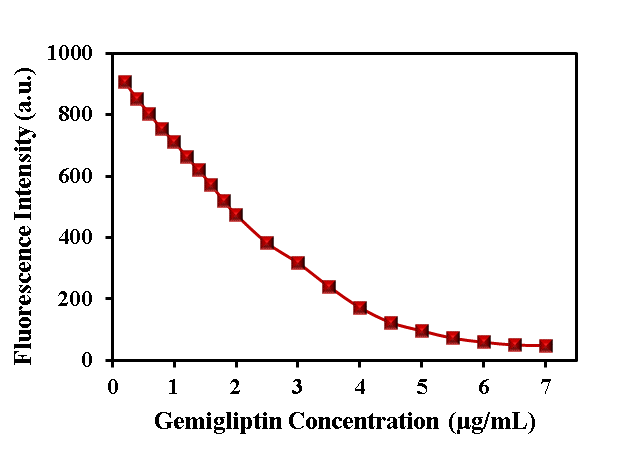

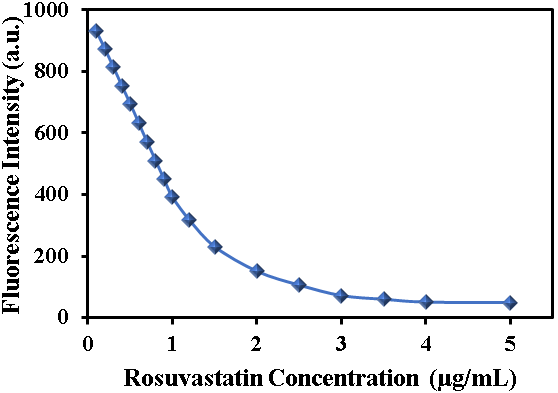


**[B]**

**[A]**

**Fig. S7.** Saturation curves of sensor **3** by addition of different concentrations of [**A**] Gemigliptin (0.2-7 μg/mL) and [**B**] Rosuvastatin (0.1-5 μg/mL).


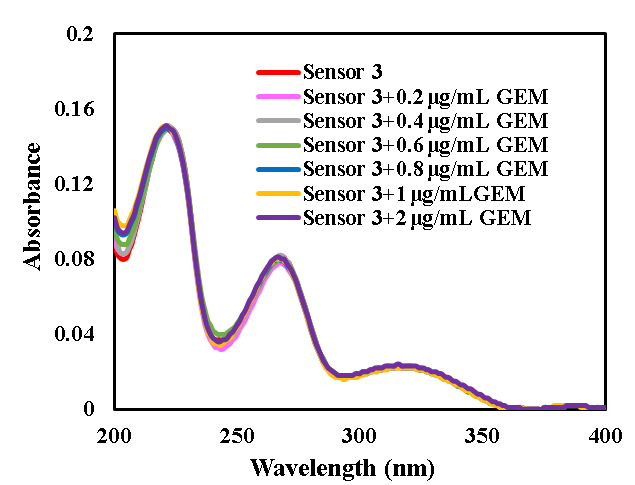

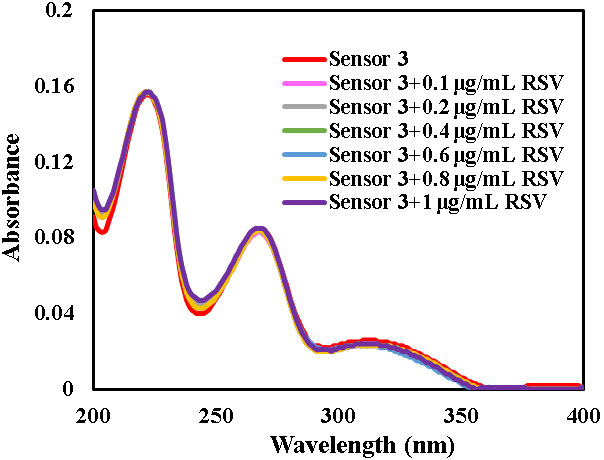


**[A]**

**[B]**

**Fig. S8.** Uv-vis spectra of sensor **3** with different concentrations of [**A**] Gemigliptin (0.2-2 μg/mL), and [**B**] Rosuvastatin (0.1-1 μg/mL) in deionized water.

**[A]**

**[B]**


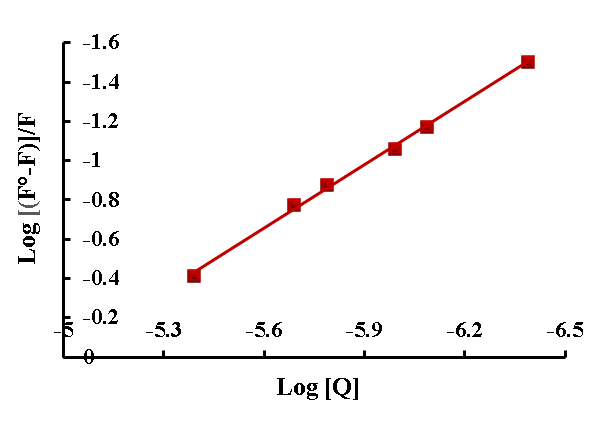

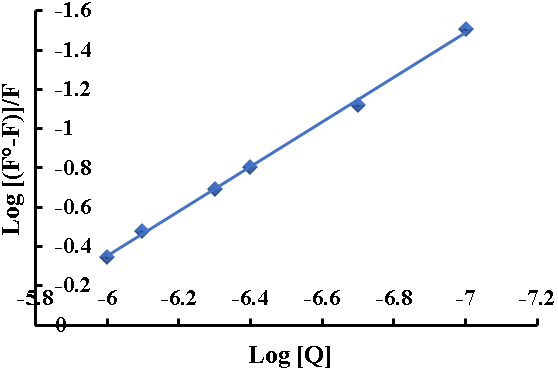


**Fig. S9.** Modified Stern-Volmer plot for the interaction of [**A**] Gemigliptin, and [**B**] Rosuvastatin with sensor **3**.

**
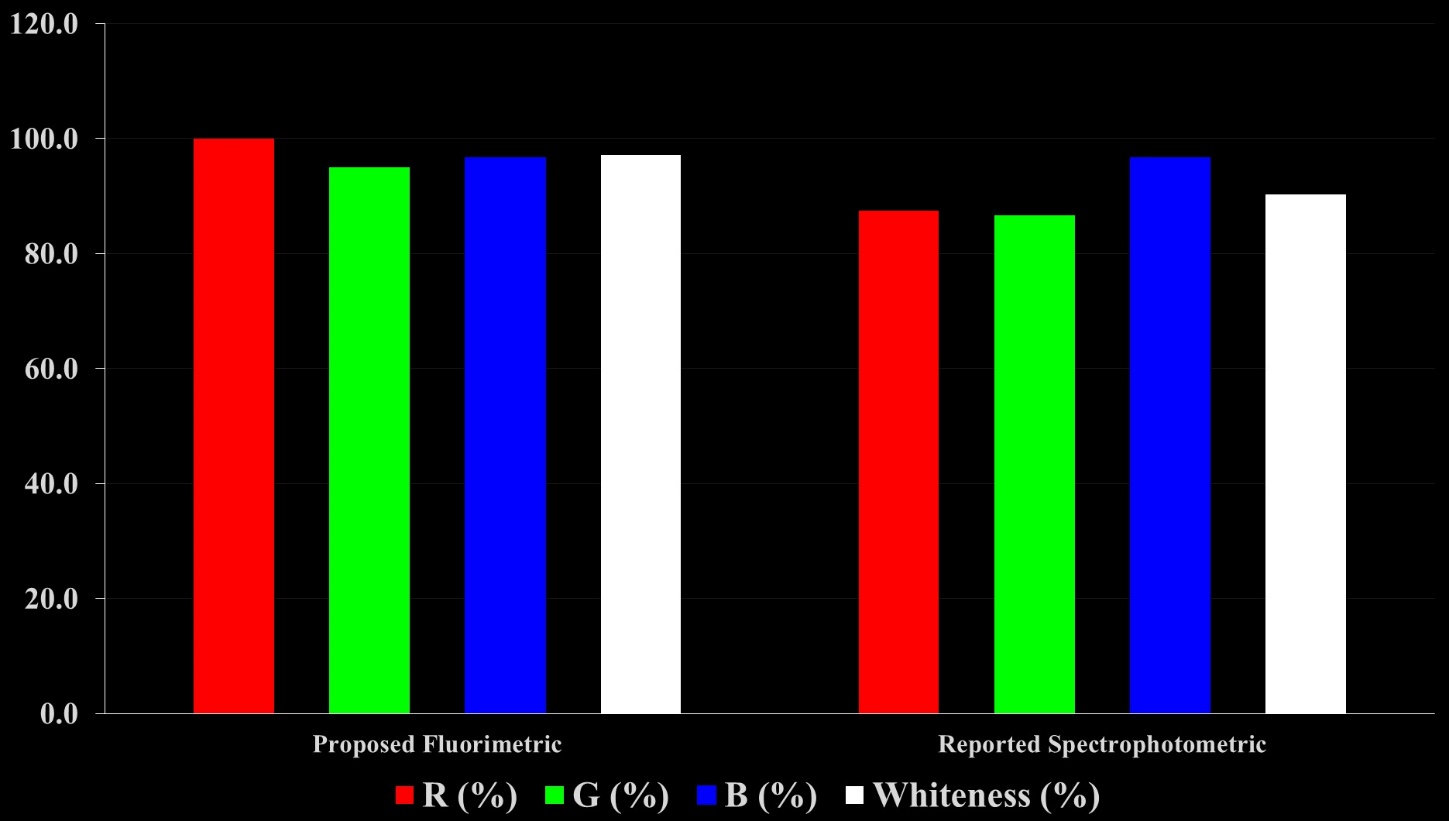
**

**Fig. S10.** The main evaluation resulted from the RGB 12 comparative assay for the adopted approach as well as the published one. The white bar reflects the mathematical average of the other three bars.

**Tables**

**Table S1.** The calculated transition energies (ΔE), wavelengths of lowest electronic transitions (λ_abs_), oscillator strengths (f), and the corresponding wavefunction configuration (weight %) were calculated with different methods for sensor **3**.

|  | **Method** | **Transition** | **ΔE (ev)** | **λabs (cal., nm)** | **f** | **λabs**  **(exp, nm)** | **Wavefunction (weight%)** |
| --- | --- | --- | --- | --- | --- | --- | --- |
| **Absorption** | **B3LYP** | S_0_ →S_1_ | 3.96 | 313 | 0.08 | 320 | H→L (96) |
|  |  | S_0_ →S_3_ | 4.90 | 253 | 0.21 | 265 | H→L+1 (89) |
|  |  | S_0_ →S_6_ | 5.60 | 221 | 0.34 | 226 | H→L+2 (41), H-3→L (39) |
|  | **MS-CASPT2** | S_0_ →S_1_ | 3.62 | 343 | 0.06 |  |  |
|  |  | S_0_ →S_3_ | 4.32 | 287 | 0.21 |  |  |
|  |  | S_0_ →S_5_ | 5.31 | 233 | 0.30 |  |  |
| **Emission** | **B3LYP** | S_1_ →S_0_ | 3.39 | 265 | 0.1 | 406 | L→H (97) |
|  | **MS-CASPT2** | S_1_ →S_0_ | 2.98 | 416 | 0.24 |  |  |

**Table S2.** Robustness assessment of the designed fluorimetric method.

| **Parameters** | **GEM** | | **RSV** | |
| --- | --- | --- | --- | --- |
|  | **Mean % recovery ± SD** | **RSD%** | **Mean % recovery ± SD** | **RSD%** |
| **Fluorophore volume (0.2 ±0.02 mL)** | ‎99.37 ± 0.78 | 0.78 | 100.02 ± 0.19 | 0.19 |
| **SDS volume (1.5 ±0.2 mL)** | 100.75 ± 0.64 | 0.64 | 99.70 ± 1.08 | 1.08 |
| **pH (6.8 ±0.2)** | 99.87 ± 0.95 | 0.95 | 99.25 ± 0.75 | 0.76 |
| **λ_ex_ (226 ±2 nm)** | 98.77 ± 1.13 | 1.14 | 98.91 ± 0.96 | 0.97 |
| **λ_em_ (406 ±2 nm)** | 99.50 ± 0.57 | 0.57 | 99.68 ± 1.26 | 1.26 |
